# Duplex formation in a G-quadruplex bulge

**DOI:** 10.1093/nar/gkaa738

**Published:** 2020-09-22

**Authors:** Thi Quynh Ngoc Nguyen, Kah Wai Lim, Anh Tuân Phan

**Affiliations:** School of Physical and Mathematical Sciences, Nanyang Technological University, Singapore 637371, Singapore; School of Physical and Mathematical Sciences, Nanyang Technological University, Singapore 637371, Singapore; School of Physical and Mathematical Sciences, Nanyang Technological University, Singapore 637371, Singapore; NTU Institute of Structural Biology, Nanyang Technological University, Singapore 636921, Singapore

## Abstract

Beyond the consensus definition of G-quadruplex-forming motifs with tracts of continuous guanines, G-quadruplexes harboring bulges in the G-tetrad core are prevalent in the human genome. Here, we study the incorporation of a duplex hairpin within a bulge of a G-quadruplex. The NMR solution structure of a G-quadruplex containing a duplex bulge was resolved, revealing the structural details of the junction between the duplex bulge and the G-quadruplex. Unexpectedly, instead of an orthogonal connection the duplex stem was observed to stack below the G-quadruplex forming a unique quadruplex–duplex junction. Breaking up of the immediate base pair step at the junction, coupled with a narrowing of the duplex groove within the context of the bulge, led to a progressive transition between the quadruplex and duplex segments. This study revealed that a duplex bulge can be formed at various positions of a G-quadruplex scaffold. In contrast to a non-structured bulge, the stability of a G-quadruplex slightly increases with an increase in the duplex bulge size. A G-quadruplex structure containing a duplex bulge of up to 33 nt in size was shown to form, which was much larger than the previously reported 7-nt bulge. With G-quadruplexes containing duplex bulges representing new structural motifs with potential biological significance, our findings would broaden the definition of potential G-quadruplex-forming sequences.

## INTRODUCTION

Guanine-rich oligonucleotides have a propensity to adopt non-canonical nucleic acid structures known as G-quadruplexes in the presence of monovalent cations, such as potassium and sodium (1–3). The G-quadruplex is a four-stranded helical structure containing multiple stacked G•G•G•G tetrads (G-tetrads), each formed by association of four guanines through Hoogsteen hydrogen bonding (4,5). G-quadruplexes can assume a variety of topologies, as defined by different combinations of strand orientations and loop configurations (6–8). There are four distinct basic core topologies, namely all-parallel-stranded, (3+1) hybrid, antiparallel-stranded up–up–down–down, and antiparallel-stranded up–down–up–down; the core strands are connected by loops, of which there are three general types: edgewise (or lateral), diagonal and double-chain-reversal (or propeller).

Over the past decades, numerous G-quadruplex motifs were found in the functional regions of the human genome (9), such as the telomeres (10–15), RNA transcripts (16–19), and promoter regions of a wide range of critical genes, e.g. *MYC* (20,21), *KIT* (22), *KRAS* (23–27), *BCL2* (28), *hTERT* (29–31), *VEGF* (32), *HIF-1α* (33), *RET* (34,35) and *PARP1* (36). There is growing evidence showing the formation of G-quadruplexes in human cells (37–39) and their regulatory roles in biological processes, including telomere length maintenance, replication, transcription, splicing and translation (40).

Most of reported G-quadruplex structures are adopted by sequences following the consensus motif G_3+_N_1–7_G_3+_N_1–7_G_3+_N_1–7_G_3+_ (41,42). However, recent studies have shown that some sequences, which do not strictly conform to this algorithm, are capable of forming stable G-quadruplexes (43–46). For example, G-quadruplex motifs from the human *MYC* promoter (47) and *KIT* promoter (48) were shown to form unusual G-quadruplex structures, which contain a G-column made of discontinuous guanine residues. Various motifs of long-loop G-quadruplex structures containing duplex stem-loops were demonstrated to assemble into quadruplex–duplex hybrids, which displayed high stability (43,45,49,50). These duplex stem-loop-containing G-quadruplex motifs were found at regulatory important regions of the human genome (51), such as the promoter region of cancer-associate genes *RAB3D* and *RAB12*, the 5′-UTR region of the *PIM1* oncogene and the intron region of myelin transcription factor. Duplex formation was shown to help drive the G-quadruplex folding process (43,45,49,52–57).

On the other hand, the formation of G-quadruplexes with bulges was reported (44). It was shown that a G-quadruplex can tolerate the presence of bulges (between adjacent guanine residues) within a G-column of the G-tetrad core, wherein increasing in the bulge size resulted in a significant decrease in the G-quadruplex stability. The formation of bulges in a G-quadruplex extended significantly the number of possible G-quadruplex-forming sequences. Recently, over 200 000 distinct bulge-containing G-quadruplex motifs were experimentally detected in the human genome by a sequencing-based method, suggesting the biological relevance of such motifs (58). Several reported bulge-containing G-quadruplex structures located at regulatory genomic regions, such as the *PARP1* promoter (36), upstream of *BCL2* promoter (59), 5′-terminal region of human telomerase RNA (hTERC) (60).

Here, we present the solution NMR structure of a G-quadruplex containing a duplex bulge, which provides the structural details of the junction between the duplex bulge and the G-quadruplex. Unexpectedly, instead of an orthogonal connection, the duplex stem was observed to stack below the G-quadruplex, forming a unique quadruplex–duplex junction. The influence of the size and position of the duplex bulge on the structure and stability of the G-quadruplex scaffold was investigated. This study revealed that a duplex bulge can be formed at various positions of a G-quadruplex scaffold. In contrast to a non-structured bulge (44), the stability of a G-quadruplex slightly increases with increasing duplex bulge size. A G-quadruplex structure containing a duplex bulge of up to 33 nt in size was shown to form. Our finding would further broaden the definition of G-quadruplex-forming sequences. These quadruplexes containing duplex bulges represent new structural duplex-quadruplex hybrid motifs with potential biological significance.

## MATERIALS AND METHODS

### DNA sample preparation

Unlabeled and site-specific ^15^N-labeled DNA oligonucleotide were synthesized on an ABI 394 DNA/RNA synthesizer using products from Glen Research and Cambridge Isotope Laboratories. The oligonucleotides were then purified following the Glen Research protocol and dialyzed successively against water, 20 mM KCl, and water again. Samples were then dried in a lyophilizer. Unless otherwise stated, the oligonucleotides were dissolved in a buffer containing 30 mM KCl and 20 mM KPi (pH 7.0).

### NMR spectroscopy

NMR experiments were performed on 600 and 700 MHz Bruker spectrometers at 25°C, unless otherwise specified. DNA samples were dissolved in a buffer containing 30 mM KCl and 20 mM KPi (pH 7). The DNA concentration of NMR samples was typically 0.2–1.0 mM. Resonances for guanine residues were assigned unambiguously using 2–4% site-specific ^15^N labeling. Spectral assignments were completed by NOESY, COSY, TOCSY and ^13^C–^1^H-HSQC experiments. Spectra were processed and analyzed by using the program FELIX (Felix NMR, Inc.).

### Structure calculation

Structures of the duplex–quadruplex construct *B4-dx2* were calculated using the program XPLOR-NIH (61). Hydrogen-bond, inter-proton distance, dihedral, planarity and repulsive restraints were imposed during structure calculations. Inter-proton distance restraints, deduced from NOESY experiments at various mixing times (100, 200 and 300 ms), were classified as very strong, strong, medium, weak and very weak. Initially, an extended conformation of the sequence was generated randomly. Structure embedding was performed to produce a family of 100 structures. Next, the 100 structures were subjected to simulated-annealing regularization and refinement. The distance constraints of exchangeable and non-exchangeable protons, G-tetrad hydrogen bonding, Watson-Crick hydrogen bonding and negative constraints were incorporated in this step of calculation. 100 structures obtained from distance-geometry simulated-annealing calculation were subjected to NOE-restrained simulated-annealing refinement in XPLOR. The system was heated up from 300 to 1000 K in 14 ps, and allowed to equilibrate for 6 ps, wherein the force constant was maintained at 2 kcal mol^−1^ Å^−2^ for exchangeable and non-exchangeable proton distance restraints, and 8 kcal mol^−1^ Å^−2^ for repulsive distance restraints. Subsequently, the force constants were scaled to 32, 16, 8 and 0 kcal mol^−1^ Å^−2^ for hydrogen-bond, non-exchangeable proton, exchangeable proton and repulsive distance restraints, respectively. Next, the system was allowed to equilibrate at 1000 K for 200 ps, then gradually cooled down to 300 K in 42 ps. After that, the system was subjected to NOE-restrained molecular dynamics calculation for 18 ps. During the last 10 ps of NOE-restrained molecular dynamics calculation, the coordinates of the systems were saved every 0.5 ps and averaged. The resulting averaged structures were submitted to Powel minimization until the energy gradient was smaller than 0.1 Kcal/mol. Dihedral (50 kcal mol^−1^ Å^−2^) and planarity (1 kcal mol^−1^ Å^−2^ for G-tetrads; 0.5 kcal mol^−1^ Å^−2^ for Watson-Crick base pairs) restraints were maintained throughout the course of refinement. Out of 100 conformers, 10 best structures with the lowest overall energy were selected. The structures were displayed using the program PyMOL (DeLano Scientific LLC).

### Circular dichroism

Circular dichroism (CD) experiments were performed on a JASCO-815 spectropolarimeter. Unless otherwise stated, CD spectra were recorded at 25°C over the range of 220−320 nm using a 1-cm path-length quartz cuvette with a reaction volume of 500 μl. The bandwidth, scan speed and data pitch were set at 2 nm, 200 nm/min and 1 nm, respectively. The oligonucleotide concentration was typically 3–5 μM. For each sample, an average of three scans was taken, the spectrum of the buffer was subtracted, and the data were zero-corrected at 320 nm. For CD melting experiments, cooling and heating were successively performed across the temperature range of 15−95°C. The CD signals were recorded at intervals of 0.5°C and at the wavelength of 263 nm. Two baselines corresponding to the completely folded (low temperature) and completely unfolded (high temperature) states were manually drawn in order to determine the fractions of folded and unfolded species during the melting/folding process. The melting temperature (*T*_m_) is defined as the temperature for which there are equal fractions of folded and unfolded species. For each sequence, the average *T_m_* value from the folding and unfolding experiments is presented. For clarity, only the folding curves are presented.

## RESULTS AND DISCUSSION

### Inclusion of a duplex hairpin within a bulge of a G-quadruplex

**Figure 1.**
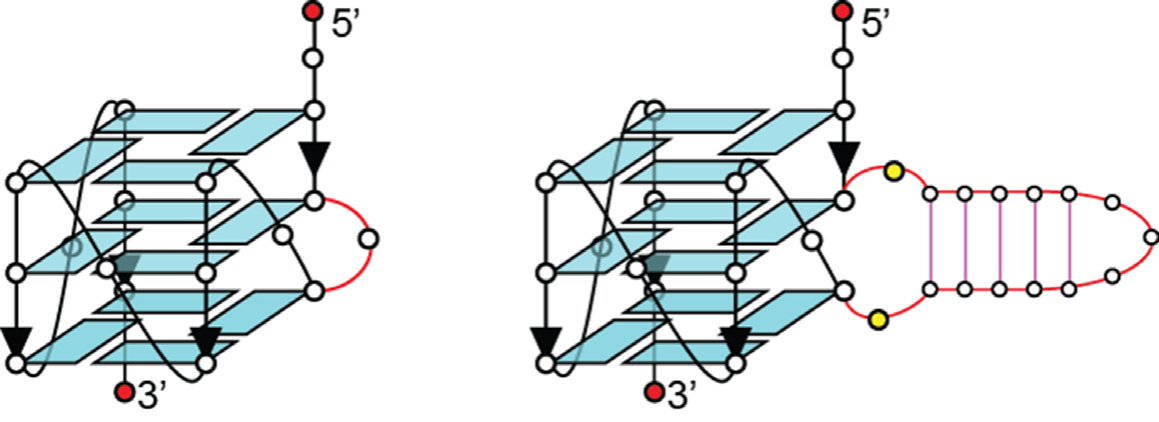
Schematics of a G-quadruplex containing a single-nucleotide bulge (left) and a duplex bulge (right).

Previously, it was shown that the sequence TTGGTGTGGGTGGGTGGGT (henceforth denoted as *B4-T*, bulge underlined; Table 1) could form a stable all-parallel-stranded G-quadruplex structure with a bulge T5 residue positioned between G4 and G6 of the first G-column (Figure 1, left) (44). To investigate the potential expansion of the bulge for an inclusion of a duplex stem, T5 was replaced by a duplex hairpin-forming motif *dx2*, giving rise to the sequence TTGG(ATCTGAGAATCAGAT)GTGGGTGGGTGGGT (henceforth denoted as *B4-dx2*; duplex hairpin *dx2* enclosed by parentheses with complementary sequences underlined; Table 1).

**Table 1.** DNA sequences used in this study

Name	Sequence^a,b^	*T*_m_(°C)^c^	Bulge size (nt)
*dx2*	ATCTGA GAA TCAGAT	-	-
*B4-T*	TT **GG**(T)**G** T **GGG** T **GGG** T **GGG** T	77 ^d^	1
*B4–15T*	TT **GG**(TTTTTTTTTTTTTTT)**G** T **GGG** T **GGG** T **GGG** T	33.5	15
*B4-dx2*	TT **GG**(ATCTGAGAATCAGAT)**G** T **GGG** T **GGG** T **GGG** T	46.2	15
*B4-dx3*	TT **GG**(ATCTGATATTAGAATAATATCAGAT)**G** T **GGG** T **GGG** T **GGG** T	49.2	25
*B4-dx4*	TT **GG**(ATCTGATATTAGCAGGAACTGCTAATATCAGAT)**G** T **GGG** T **GGG** T **GGG** T	50.5	33
*B3-dx2*	TT **G**(ATCTGAGAATCAGAT)**GG** T **GGG** T **GGG** T **GGG** T	54.5	15
*B7-dx2*	TT **GGG** T **G**(ATCTGAGAATCAGAT)**GG** T **GGG** T **GGG** T	40.9	15
*B8-dx2*	TT **GGG** T **GG**(ATCTGAGAATCAGAT)**G** T **GGG** T **GGG** T	47.2	15
*B12-dx2*	TT **GGG** T **GGG** T **GG**(ATCTGAGAATCAGAT)**G** T **GGG** T	45.6	15

^a^G-tracts are shown in bold-face.

^b^Individual bulges are enclosed by parentheses with complementary tracts underlined.

^c^The meting temperatures (*T*_m_) of samples in a buffer containing 30 mM KCl and 20 mM KPi (pH 7) were monitored by the CD signals at 263 nm.

^d^ *T*_m_ was obtained from reference (44).

**Figure 2.**
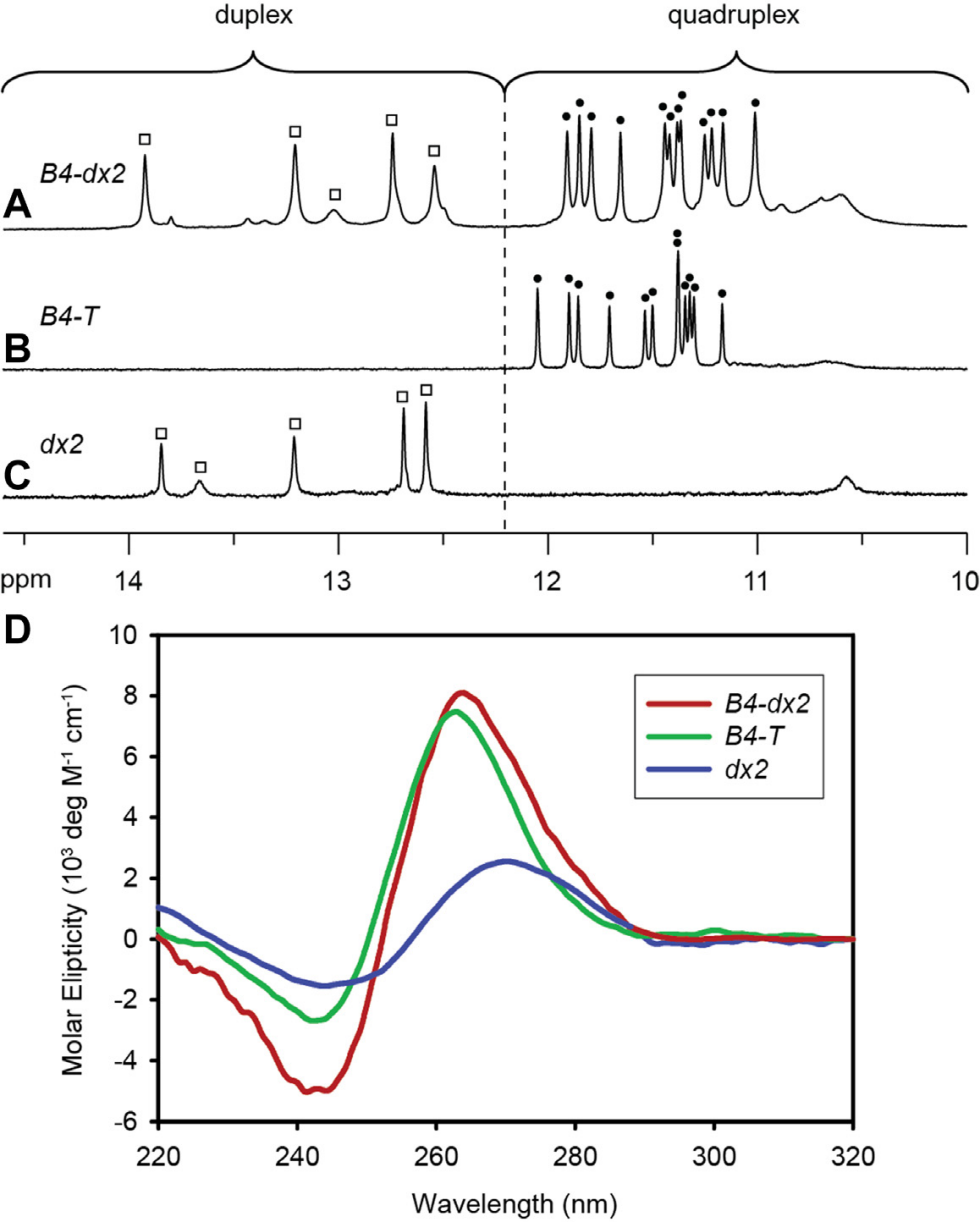
1D imino proton NMR spectra of (**A**) *B4-dx2*, (**B**) *B4-T* and (**C**) *dx2*. Imino protons of the G-tetrad core are indicated by filled circles, whereas imino protons of the duplex are indicated by open squares. (**D**) CD spectra of *B4-T* (green), *dx2* (blue) and *B4-dx2* (red).

One-dimensional (1D) imino proton NMR spectrum of *B4-dx2* showed twelve major peaks at ∼10.5–12 ppm (marked by filled circles, Figure 2A), characteristic of guanine imino protons involved in the formation of G-tetrads. Five additional imino proton peaks were observed at ∼12.5–14 ppm (marked by open squares, Figure 2A), indicative of the duplex hairpin formation. Distribution patterns of these imino proton peaks closely resembled those of the individual components, G-quadruplex (*B4-T*; Figure 2B) and duplex hairpin (*dx2*; Figure 2C), indicative of the formation of a quadruplex–duplex hybrid with a duplex hairpin located within the bulge of the all-parallel-stranded G-quadruplex scaffold (Figure 1, right). CD spectrum of *B4-dx2* showed an intense positive peak at ∼260 nm and a negative trough at ∼240 nm (Figure 2D), supporting the formation of an all-parallel-stranded G-quadruplex (62–64). Overall, these observations corroborated the quadruplex–duplex hybrid nature of *B4-dx2*.

### NMR spectral assignments of *B4-dx2*

We proceeded to elucidate the solution structure of *B4-dx2*. Guanine imino and aromatic protons were unambiguously assigned (Supplementary Figure S1) through site-specific ^15^N-labeled (4%) samples (65). Spectral assignment of proton resonances of *B4-dx2* was completed by using through-bond (COSY and TOCSY) and through-space (NOESY) NMR experiments. The imino proton of the isolated guanine G20 was clearly detected at the region of 10.5–12 ppm (Supplementary Figure S1), reflecting its participation in the G-tetrad formation. Other 11 guanines involved in G-tetrads are G3–G4, G22–G24, G26–G28 and G30–G32.

### Folding topology of *B4-dx2*

**Figure 3.**
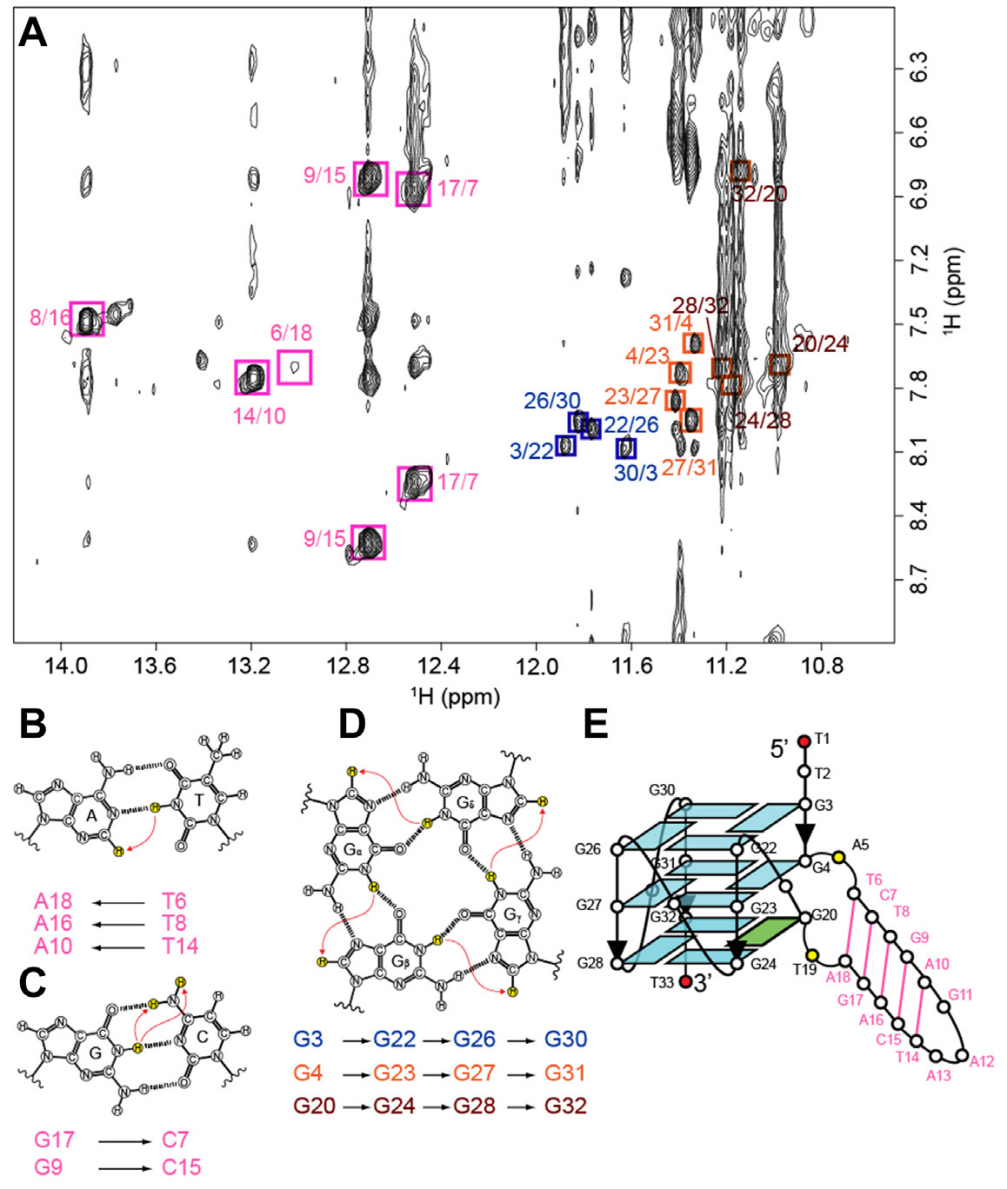
NMR structural characterization of *B4-dx2*. (**A**) NOESY spectrum (mixing time, 200 ms) showing cross-peaks for the identification of three G-tetrads and five Watson-Crick base pairs. Signature NOE cross-peaks for the Watson-Crick base pairs and G-tetrads are framed and labeled with the residue numbers of the imino and H8 protons at the first and second positions, respectively. (**B**) NOE between the thymine imino proton and the adenine H2 proton in an A•T base pair. (**C**) NOEs between the guanine imino proton and the cytosine amino protons in a G•C base pair. (**D**) NOEs between H8 and imino protons within a G-tetrad. (**E**) Schematic diagram of *B4-dx2*.

Within a G-tetrad layer, the imino proton of a guanine is in proximity of the H8 proton of the adjacent guanine and therefore would exhibit a NOE cross-peak. By observing such cross-peaks, the folding topology of a G-quadruplex can be defined (65). The NOEs of G3(H1)–G22(H8), G22(H1)–G26(H8), G26(H1)–G30(H8) and G30(H1)–G3(H8) (Figure 3A) pointed to the formation of the tetrad plane of G3•G22•G26•G30. Similarly, the other two tetrads, G4•G23•G27•G31 and G20•G24•G28•G32, were defined (Figure 3D).

Signature NOE cross-peaks can be observed in Watson–Crick base pairs due to the proximity between respective protons: NOE between the thymine imino proton and the adenine H2 proton in an A•T base pair (Figure 3A,B), as well as NOEs between the guanine imino proton and the cytosine amino protons in a G•C base pair (Figure 3A, C). These signature NOE cross-peaks were observed for five base pairs (T6•A18, G17•C7, T8•A16, G9•C15 and T14•A10), pointing to the formation of the duplex stem (Figure 3E).

The formation and arrangement of three G-tetrads and five Watson–Crick base pairs validated the topology of *B4-dx2* as an all-parallel-stranded G-quadruplex involving three continuous tracts of guanines: G22–G24, G26–G28, G30–G31 and one discontinuous tract: G3, G4 and G20 (Figure 3E). This discontinuous tract results in a bulge between G4 and G20, forming a 5-bp duplex stem. Four G-tracts are connected by three 1-nt double-chain-reversal loops: T21, T25, and T29. NOEs observed between the protons of G20 and those of G4, e.g. G20(H8)–G4(H8), G20(H8)–G4(H1′), G20(H8)–G4(H2′/H2″), G20(H8)–G4(H3′), G20(H1′)–G4(H1′) and G20(H1)–G4(H1), support the position of G20 in the G-tetrad core below G4. The typical NOE sequential connectivities (*n*)H8 and (*n* – 1)H1′/H2′/H2″/H3′/H4′ are clearly observed along the G-columns and two strands of the duplex hairpin (Supplementary Figure S2). These NOE sequential connectivities are broken at the transition point from the first G-column to the bulge and at the three double-chain-reversal loops (T21, T25 and T29). Moderate intensities of the intra-residue H8–H1′ NOE cross-peaks reflect *anti* glycosidic conformations for all guanine residues except G20, which adopts a *syn* conformation as seen by its strong intra-residue H8–H1′ NOE cross-peak.

### Solution structure of *B4-dx2*

**Figure 4.**
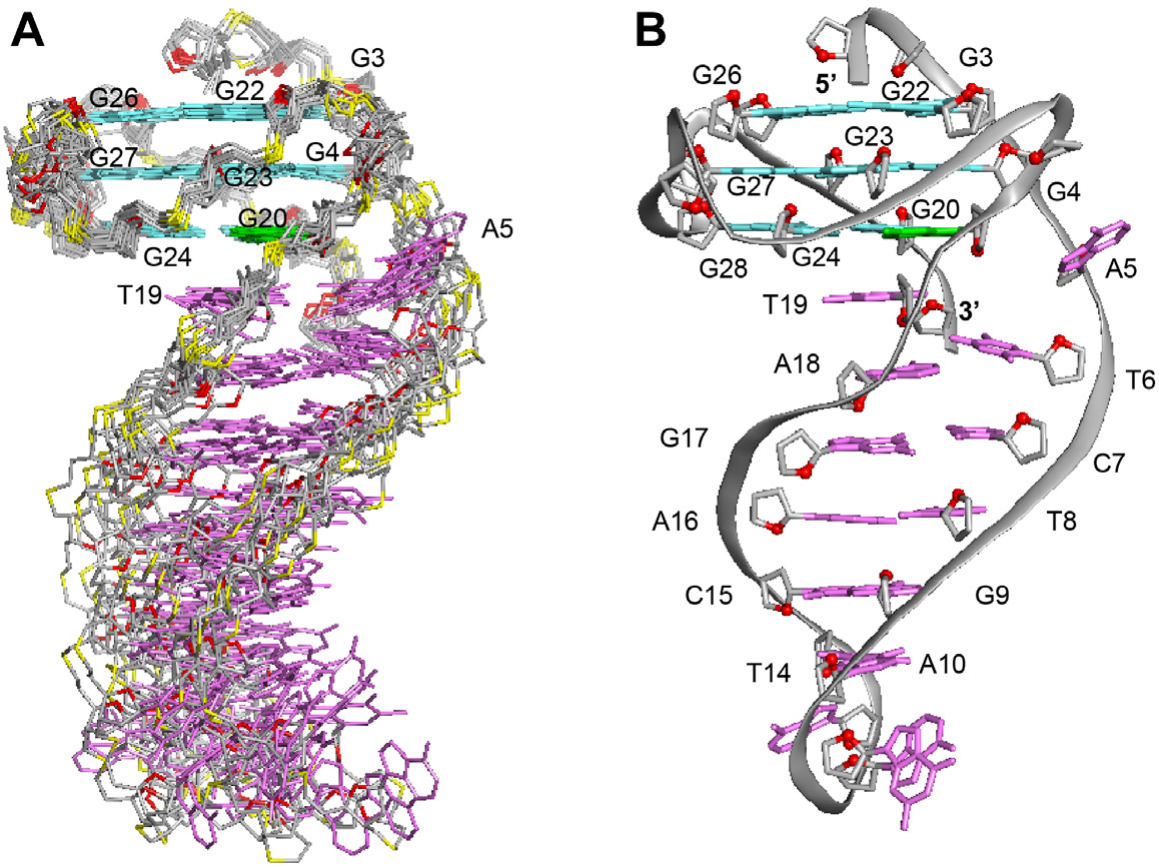
NMR solution structure of *B4-dx2*. (**A**) Ten superimposed lowest-energy structures. (**B**) Ribbon view of a representative structure. *Anti* guanines in the G-tetrads are colored in cyan; *syn* guanine in the G-tetrads, green; bases in the duplex stem, magenta; backbone and sugar, gray; O4′ atoms, red; phosphorus atoms, yellow.

The structure of *B4-dx2* (Figure 4) was calculated on the basis of NMR restraints (Table 2) using the XPLOR-NIH program (61). The G-tetrad core of *B4-dx2* adopts a three-layered all-parallel-stranded scaffold, with three single-nucleotide double-chain-reversal loops (T21, T25 and T29). The bases of these loops project outwards from the G-tetrad core.

**Table 2.** Statistics of the computed structures of *B4-dx2*

A. NMR Restraint		
Distance restraints	D_2_O	H_2_O
Intraresidue	508	9
Sequential (*i*, *i*+1)	194	17
Long-range (*i*, ≥*i*+2)	25	46
Other restraints		
Hydrogen-bond	72	
Dihedral	22	
Repulsive	15	
B. Structure statistics		
NOE violation		
Number (>0.2 Å)	0.500 ± 0.972	
Maximum violation (Å)	0.189 ± 0.063	
Rmsd of violation (Å)	0.015 ± 0.002	
Deviations from the ideal covalent geometry		
Bond length (Å)	0.003 ± 0.000	
Bond angles (°)	0.686 ± 0.009	
Impropers (°)	0.340 ± 0.006	
Pairwise all heavy atom rmsd (Å)		
All heavy atoms in G-tetrad core	0.486 ± 0.120	
All heavy atoms in duplex hairpin	0.578 ± 0.154	
All heavy atoms	1.086 ± 0.269	

**Figure 5.**
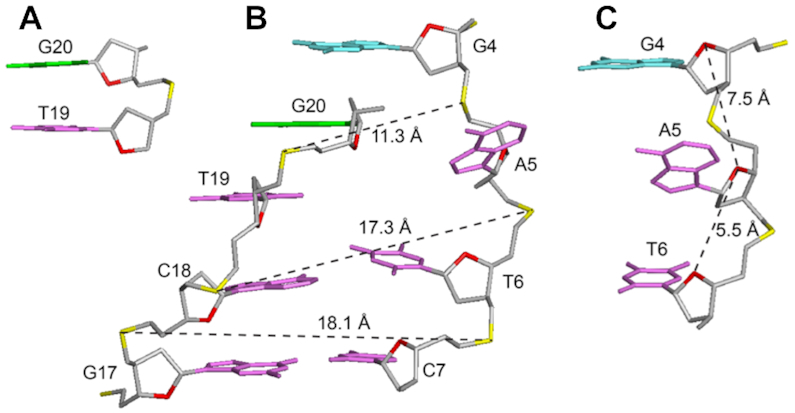
Structural features of *B4-dx2* at the quadruplex–duplex junction. (**A**) Stacking between T19 (magenta) and G20 (green). (**B**) Backbone strand separation at the junction for the duplex stem and the G-tetrad core (**C**) Stretching of backbone between the G4 and A5 residues.

The first two thymine residues (T1 and T2) are positioned on top of the G-tetrad core; the last thymine T33 is stacked below the bottom G-tetrad. The duplex at the bulge formed between G4 and G20 includes five Watson–Crick base pairs, while the immediate possible base pair step at the quadruplex–duplex junction, A5–T19, is broken up (Figure 5B). The duplex hairpin shows continuous stacking under the 3′-end G-tetrad, with T19 directly sandwiched between the tetrad and duplex while the base of A5 projects outward from the groove. Stacking between the duplex and the quadruplex is reflected by the generally upfield-shifted imino proton peaks of the G-tetrad core of *B4-dx2* (Figure 2A) as compared to those of *B4-T* (Figure 2B) in the region of 11–12 ppm. The placement of T19 right below the 6-membered ring of G20 of the G-tetrad (Figure 5A) is consistent with the up-field shift of resonances belonging to T19 and G20. Stacking between T19 and G20 is supported by the following observed NOEs: T19(H2′/H2″)–G20(H1), T19(H1′)–G20(H1), T19(CH_3_)–G20(H1), T19(CH_3_)–G24(H1), T19(CH_3_)–G24(H8) and T19(H6)–G24(H8). To accommodate a duplex hairpin insertion within the context of a quadruplex bulge, the strand separation (defined as the distance between matching pairs of phosphate groups) at the A5-T19 step is narrower (11.3 Å), as compared to the next T6-A18 step (17.3 Å) and a normal Watson-Crick base pair step (∼18 Å) within the duplex stem (Figure 5B). Moreover, the stepwise O4′-to-O4′ distance between G4 and A5 at the junction was found to be 7.5 Å, which is considerably more stretched as compared to that observed within a duplex (5–6 Å) (Figure 5C). This configuration of A5 permits a progressive helical transition between the quadruplex and duplex segments, thus maximizing the stability of the overall structure.

Previously, various structural configurations have been observed at the quadruplex–duplex junction to bridge the transition between the two distinct helical segments. A duplex stem, when placed in different loops, can be connected to a quadruplex in either orthogonal or co-axial orientation (43). In the former case, the base pair step at the junction was found to flip open, while in the latter case continuous stacking between G-tetrads and base pairs in the duplex was observed (Supplementary Figure S3). On one hand, the duplex connection to a bulge resembles orthogonal connection, leading to the broken first base pair step. On the other hand, in *B4-dx2* only A5 was projected outward from the overall helix and continuous base stacking between the quadruplex and duplex was observed. Across other quadruplex–duplex hybrid structures, various adaptor base pairs or structural elements have been observed to bridge the two helical domains (Supplementary Figure S4) (43,50,66,67).

### Effects of the bulge size on the structure and stability of a G-quadruplex containing a duplex bulge

**Figure 6.**
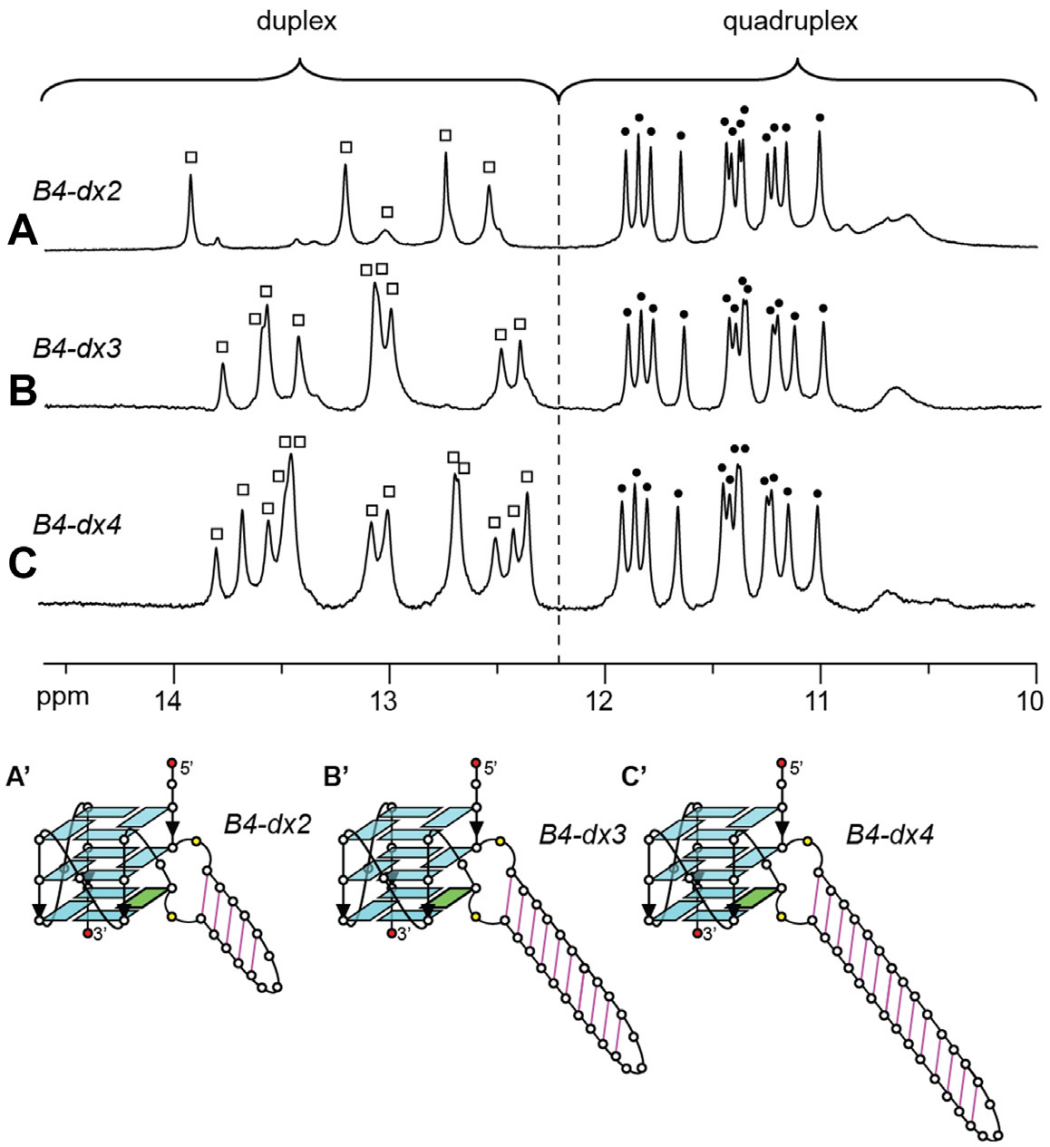
1D imino proton NMR spectra and schematics of G-quadruplexes containing a duplex bulge of different sizes: (**A**, A’) *B4-dx2* (15 nt), (**B**, B’) *B4-dx3* (25 nt) and (**C**, C’) *B4-dx4* (33 nt).

To explore the effects of larger duplex bulges on the structure and stability of a G-quadruplex scaffold, we investigated similar sequences containing duplex bulges of 25 and 33 nt (denoted as *B4-dx3* and *B4-dx4*, respectively; Table 1). In 1D imino proton NMR spectra of *B4-dx2*, *B4-dx3* and *B4-dx4*, twelve major peaks were observed at the 10.5–12 ppm region for all sequences, while the number of peaks at the 12–14 ppm region increased with increasing duplex stem length (Figure 6). The similarity of spectral patterns for *B4-dx2*, *B4-dx3* and *B4-dx4* in the region of 10.5–12 ppm suggested the formation of the same G-quadruplex scaffold. CD spectra of these three structures also showed the signature of an all-parallel-stranded G-quadruplex structure with a positive peak at ∼260 nm and a negative trough at ∼240 nm (Supplementary Figure S5).

These structures exhibited a high thermal stability, with the melting temperature (*T*_m_) ranging from 46 to 50.5°C in the presence of ∼60 mM K^+^ (Table 1). Previously, it has been shown that increasing the bulge size led to decreasing stability of the G-quadruplex (44). In this study, as observed in the case of *B4-dx2*, *B4-dx3* and *B4-dx4*, increasing the size of a duplex bulge did not lead to a decrease in the stability of the G-quadruplex. On the contrary, the melting temperature slightly increased from 46.2°C to 50.5°C, as the duplex bulge size was increased from 15 to 33 nt (Supplementary Figure S6). A similar trend was also observed when a duplex of increasing size was incorporated within a G-quadruplex loop (49). The melting temperature of a G-quadruplex was observed to increase by ∼13°C (Supplementary Figure S6) when a non-structured bulge (*B4–15T*; Table 1) is replaced by a duplex bulge with the same length (*B4-dx2*), indicating that a G-quadruplex with a duplex bulge exhibited significantly higher thermal stability than a G-quadruplex containing a non-structured bulge of the same length.

### Insertion of a duplex bulge at different positions of a G-quadruplex

**Figure 7.**
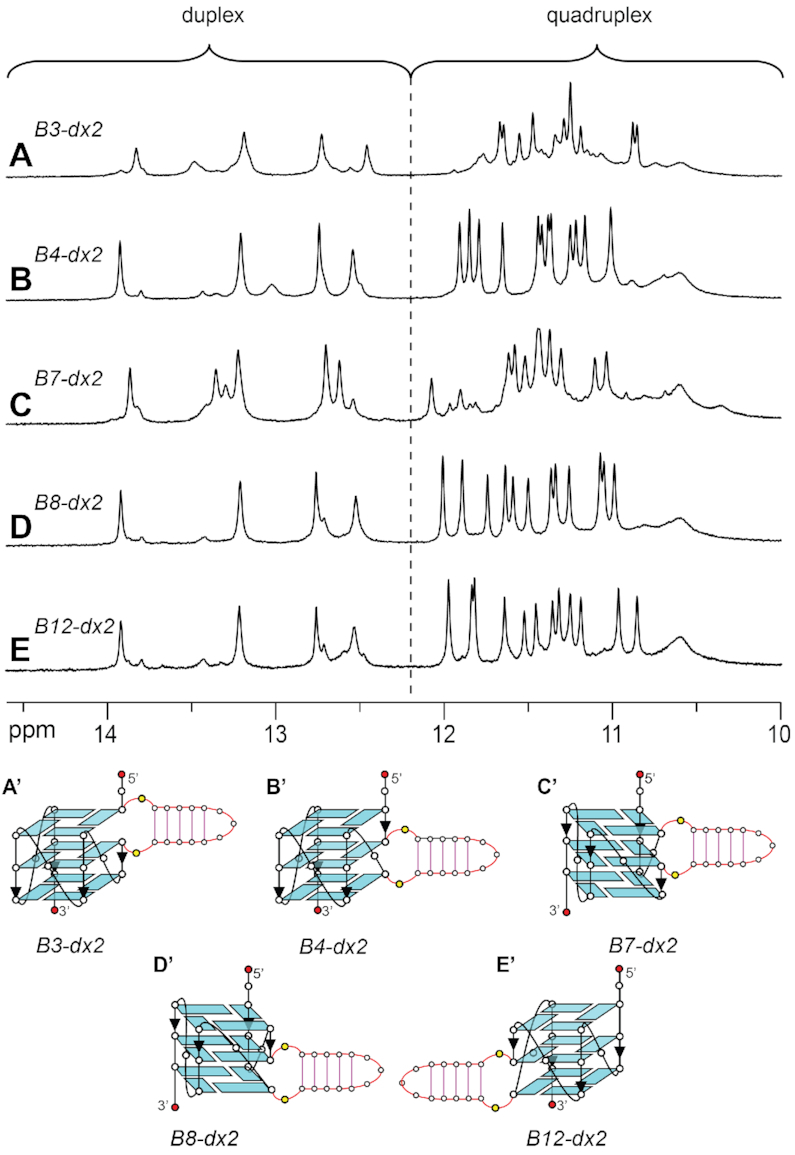
1D imino proton NMR spectra and schematics of quadruplex–duplex hybrids with a duplex hairpin inserted at different bulge positions: (**A**, A’) *B3-dx2* with duplex at the bulge position 3, (**B**, B’) *B4-dx2* with duplex at the bulge position 4, (**C**, C’) *B7-dx2* with duplex at the bulge position 7, (**D**, D’) *B8-dx2* with duplex at the bulge position 8, and (**E**, E’) *B12-dx2* with duplex at the bulge position 12.

To investigate the effect of different placements of a duplex bulge on the structure and stability of a G-quadruplex scaffold, the duplex hairpin *dx2* was inserted at various bulge positions within the G-tetrad core (sequences *B3-dx2*, *B4-dx2*, *B7-dx2*, *B8-dx2* and *B12-dx2*, Table 1). Similar CD spectra, showing a positive peak at ∼260 nm and a negative peak at ∼240 nm, were observed for all five sequences (Supplementary Figure S7), suggesting that these sequences adopt an all-parallel-stranded G-quadruplex fold. In all cases, imino proton peaks were observed in both G-quadruplex (10.5–12 ppm) and duplex (12–14 ppm) characteristic regions (Figure 7), supporting the formation of G-quadruplex–duplex hybrids. In the cases of *B4-dx2*, *B8-dx2* and *B12-dx2*, a major conformation with similar spectral patterns was observed, while in the cases of *B3-dx2* and *B7-dx2*, significant minor conformations were also observed. The stability of these structures was found to depend on the position of the duplex bulge, with *T*_m_ ranging from 40.9°C to 54.5°C in the presence of ∼60 mM K^+^ (Table 1).

### Broadening the definition of G-quadruplex forming sequences

It was previously shown that sequences with disrupted G-tracts can form G-quadruplexes with bulges of a size ranging from 1 to 7 nt (44). Among over 700 000 G-quadruplexes experimentally detected in the human genome, over 200 000 of which were G-quadruplexes with bulges (58). In this study, we have shown that the size of a bulge can be extended to 33 nt or even longer if base pairs are formed within the bulge. Such a duplex bulge can exist at different positions within a G-quadruplex scaffold, significantly increasing the space of G-quadruplex-forming sequences beyond the standard G_3+_N_1–7_G_3+_N_1–7_G_3+_N_1–7_G_3+_ motif previously defined for potential G-quadruplex-forming sequences (41,42). This has broadened the definition of G-quadruplex-forming sequences and can greatly increase the number of potential G-quadruplex-forming sites in the genome.

### A unique structure for specific G-quadruplex targeting

Targeting G-quadruplexes was shown to be a promising strategy for anti-cancer therapy (68). Numerous G-quadruplex-binding ligands have been developed with the current generation of ligands exhibiting high affinity toward G-quadruplexes and high selectivity between G-quadruplex and other DNA structures (69–79). However, these ligands still show poor selectivity between different G-quadruplex structures. As most G-quadruplex-binding ligands stack on the top and bottom G-tetrads, it is challenging to achieve specific targeting of a single G-quadruplex amongst over 700 000 potential G-quadruplex structures across the human genome (58). There has been increasing interest in designing specific G-quadruplex-binding ligands, where additional structural features besides the G-tetrad core, such as loops and bulges, could provide insights for the design of highly specific ligands. The G-quadruplex structure with a duplex bulge in this study presents a new type of quadruplex–duplex hybrid which can serve as a unique structure for specific G-quadruplex targeting. Such a motif was identified in the *BCL2* proximal promoter (59), suggesting its biological relevance and therapeutic potential. The BCL2 protein plays an important role in the regulation of programed cell death and its overexpression is correlated with a large number of cancers. There are two GC-rich regions identified in the upstream of the *P1* promoter of *BCL2* (denoted as *Pu39* and *P1G4*) (59). The major G-quadruplex form adopted by *Pu39*, which was shown to be a *BCL2* transcription activator, was a parallel G-quadruplex with one 13-nt and two 1-nt unstructured loops (80). On the other hand, *P1G4* can form two interchangeable parallel G-quadruplex conformers: a duplex stem-loop G-quadruplex and a duplex stem-bulge G-quadruplex, which were shown to be transcription repressors (59). *BCL2* contains multiple transcription start sites and the formation of *Pu39* or *P1G4* G-quadruplexes could be correlated with the transcription start sites being used (59). The existence of a duplex stem in the *P1G4* G-quadruplexes could provide a unique feature for these G-quadruplexes as compared to *Pu39* or other G-quadruplexes for the binding of protein or small molecule to precisely regulate the transcription of *BCL2*. Unique structural features of a G-quadruplex with a duplex bulge, such as a broken G-column induced by a bulge, can allow the intercalation of a ligand, while the minor groove of a duplex bulge can serve as a substrate for specific targeting by ligands with duplex sequence selectivity (81–84). Pyrrole-imidazole polyamides, for example, can be programmed to target a duplex sequence with high selectivity (85,86). Highly specific targeting of a G-quadruplex structure containing a duplex bulge can be achieved by simultaneous targeting the duplex part, the G-quadruplex part and the junction between the duplex and the G-quadruplex.

## CONCLUSION

In this study, we showed that a duplex stem can be incorporated into a bulge of a G-quadruplex. The NMR solution structure of a G-quadruplex containing a duplex bulge was presented, showing a unique quadruplex–duplex junction with the duplex bulge stacking below the 3′-end G-tetrad. Breaking up of the immediate base pair step at the quadruplex–duplex junction, coupled with a narrowing of the duplex groove within the context of the bulge, led to a progressive transition between the quadruplex and duplex segments. Duplex bulges can occur at various positions of a G-quadruplex scaffold and increasing the length of a duplex bulge does not lead to a decrease in the stability of the overall G-quadruplex core. The formation of a long duplex bulge within G-quadruplexes would expand the current knowledge on predicting G-quadruplex-forming sequences. Potential existence of such structures in the human genome may serve as unique targets for designing ligands for specific G-quadruplex targeting.

## DATA AVAILABILITY

The coordinates for the NMR solution structure of *B4-dx2* have been deposited in the Protein Data Bank (PDB ID: 7CLS).

## Supplementary Material

gkaa738_Supplemental_File
