# Supplementary material for: Duplex formation in a G-quadruplex bulge
Source: Nucleic Acids Res. 2020 Sep 22;48(18):10567–75. doi: 10.1093/nar/gkaa738 (PMC7544226; doi:10.1093/nar/gkaa738)
Supplement: gkaa738_Supplemental_File [file gkaa738_supplemental_file.pdf]

Duplex formation in a G-quadruplex bulge

Thi Quynh Ngoc Nguyen[1], Kah Wai Lim[1], and Anh Tuân Phan[1,2,*]

[1] School of Physical and Mathematical Sciences, Nanyang Technological University, Singapore 637371, Singapore

[2] NTU Institute of Structural Biology, Nanyang Technological University, Singapore 636921, Singapore

Supplementary Data

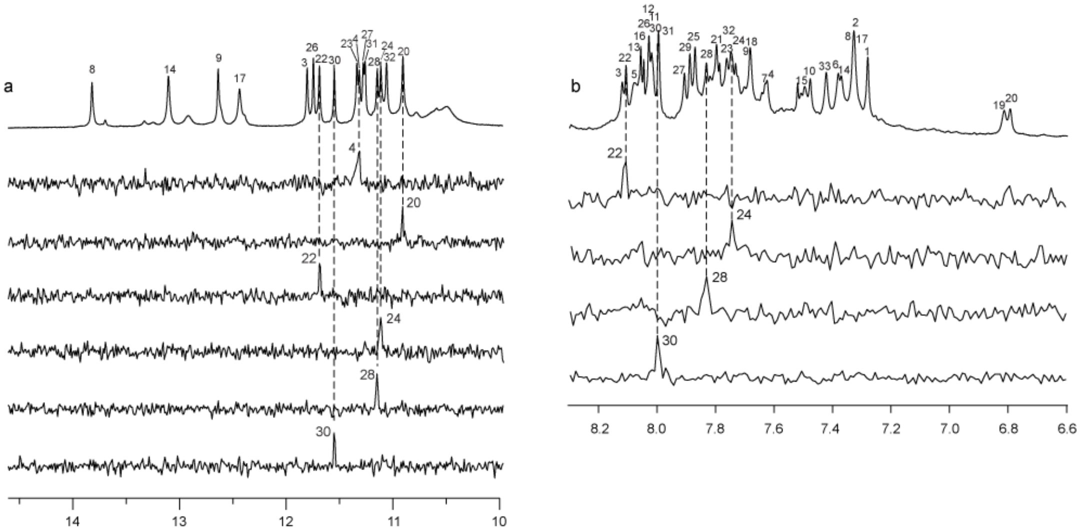

Figure S1. Assignments of guanine imino and aromatic protons of *B4-dx2* in K^+ solution using 4% ^{15}N-labeled samples: (a) Guanine imino protons; (b) guanine H8 protons.

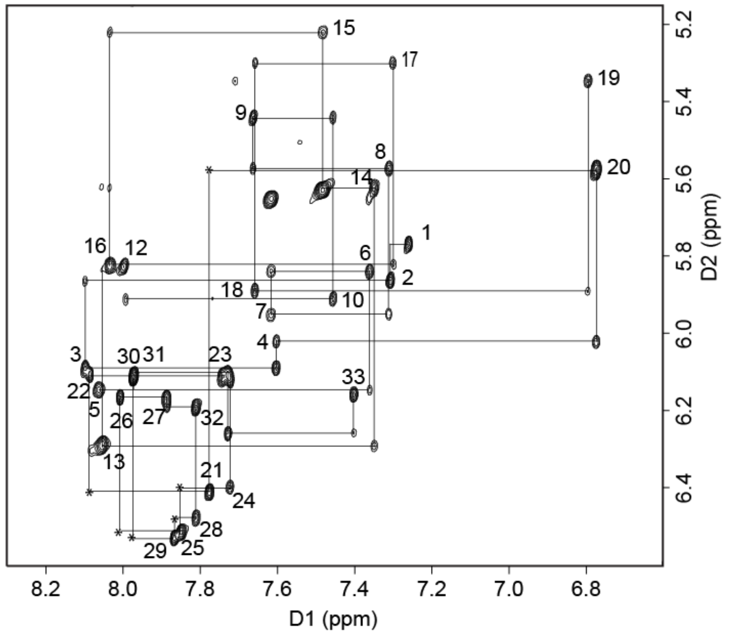

Figure S2. NOESY spectrum (mixing time, 300 ms) of *B4-dx2* in K^+ solution showing H8/H6-H1' connectivity. Intra-residue H8/H6-H1' cross peaks are labeled with their respective residue numbers.

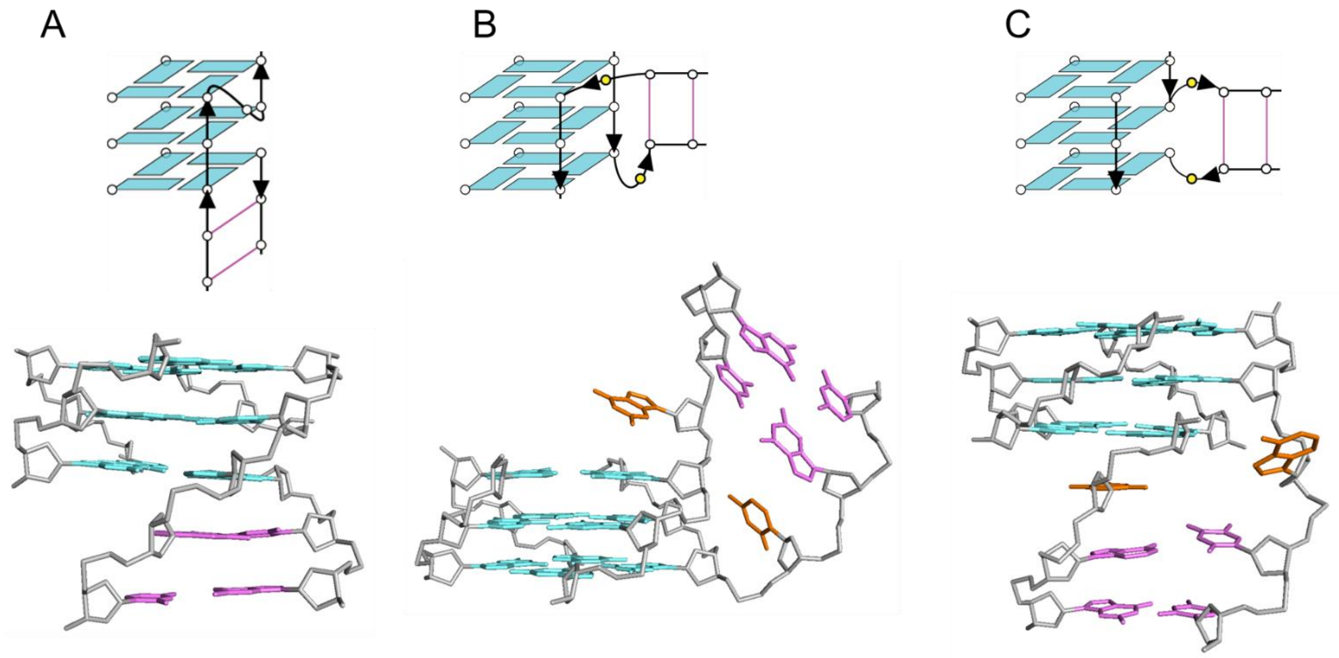

Figure S3. Quadruplex-duplex connection in (A) Coaxial connection (PDB: 2M90) (1); (B) Orthogonal connection (PDB: 2M93) (1); (C) Duplex bulge connection (PDB: 7CLS). The schematics are shown at the top, the atomic structures are shown at the bottom. Guanines in G-tetrads are colored in cyan; bases in duplex stem, magenta; bases at G-quadruplex-duplex junctions, orange; backbone and sugar, gray.

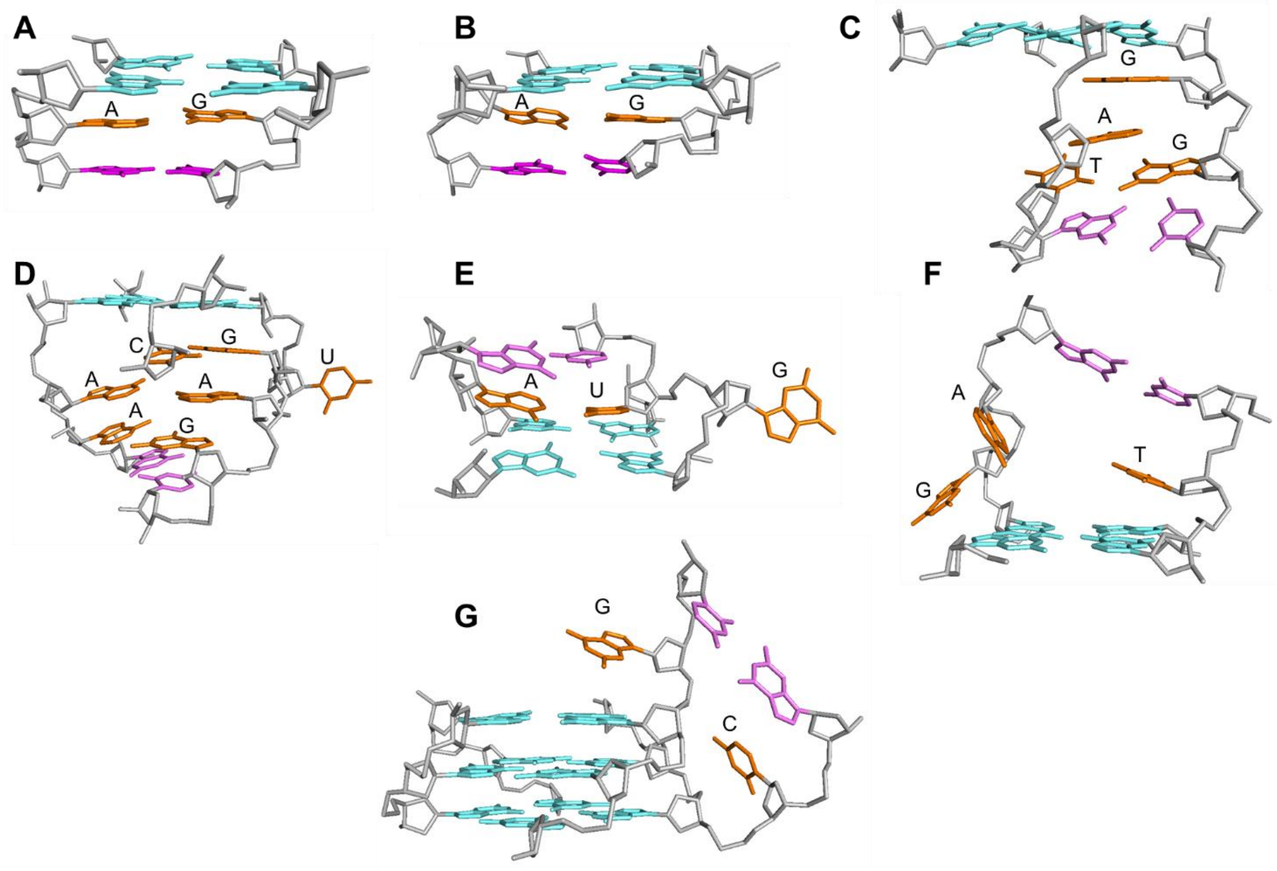

Figure S4. Quadruplex-duplex junctions with various adaptors from reported quadruplex-duplex hybrids: (A) PDB code: 2M91 (1); (B) PDB code: 2M92 (1); (C) PDB code: 5CMX (2); (D, E) PDB code: 4KZE (3); (F) PDB code: 6H1K (4); (G) PDB code: 2M93 (1). Guanines in G-tetrads are colored in cyan; bases in duplex stem, magenta; bases at G-quadruplex-duplex junctions, orange; backbone and sugar, gray.

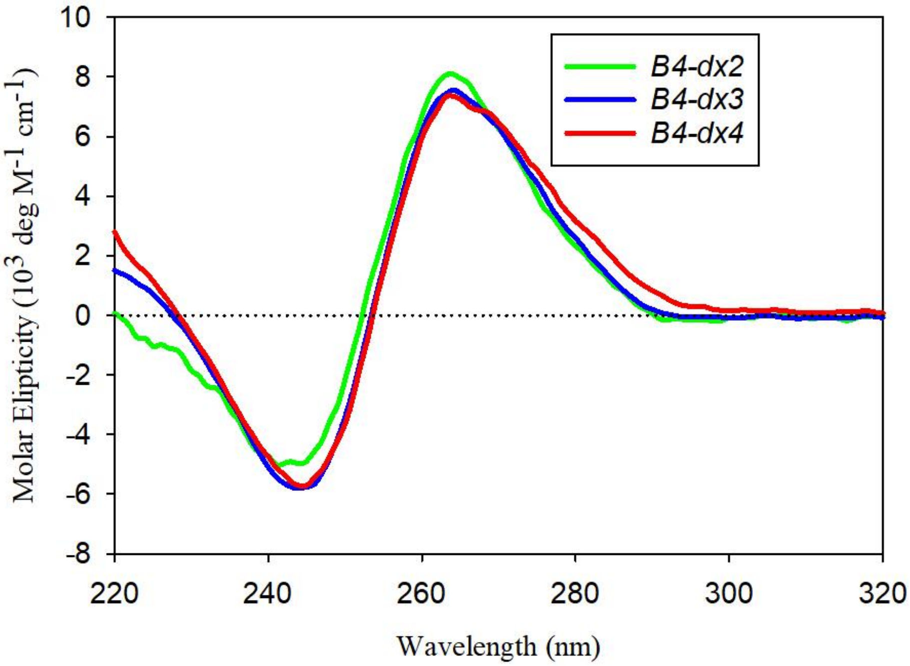

Figure S5. CD spectra of *B4-dx2* (green), *B4-dx3* (blue) and *B4-dx4* (red) in buffer containing 30 mM KCl and 20 mM KPi (pH7).

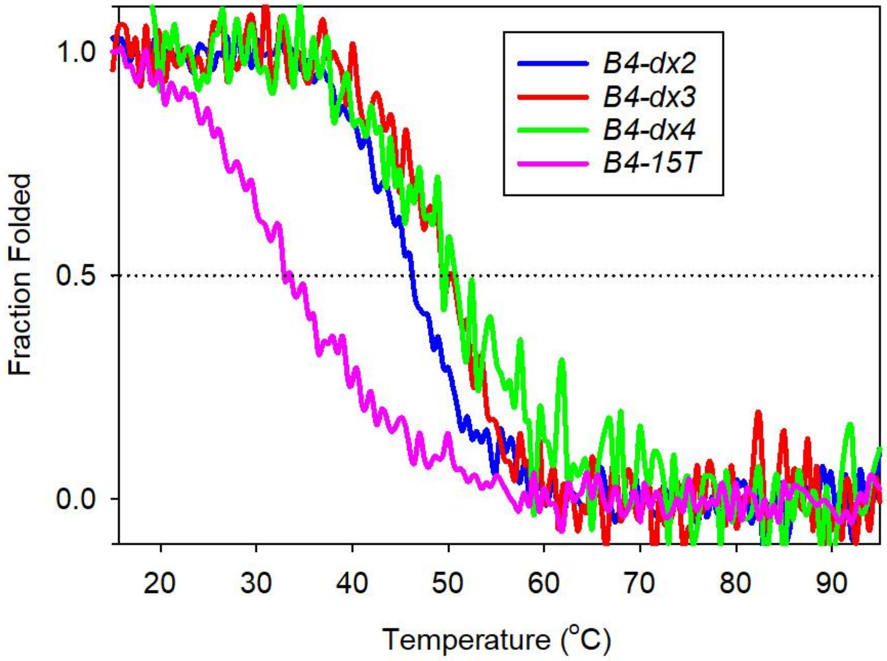

Figure S6. CD melting of G-quadruplexes containing duplex bulges of different size: *B4-dx2* (blue), *B4-dx3* (red), *B4-dx4* (green), and the reference *B4-15T* (pink) in buffer containing 30 mM KCl and 20 mM KPi (pH 7).

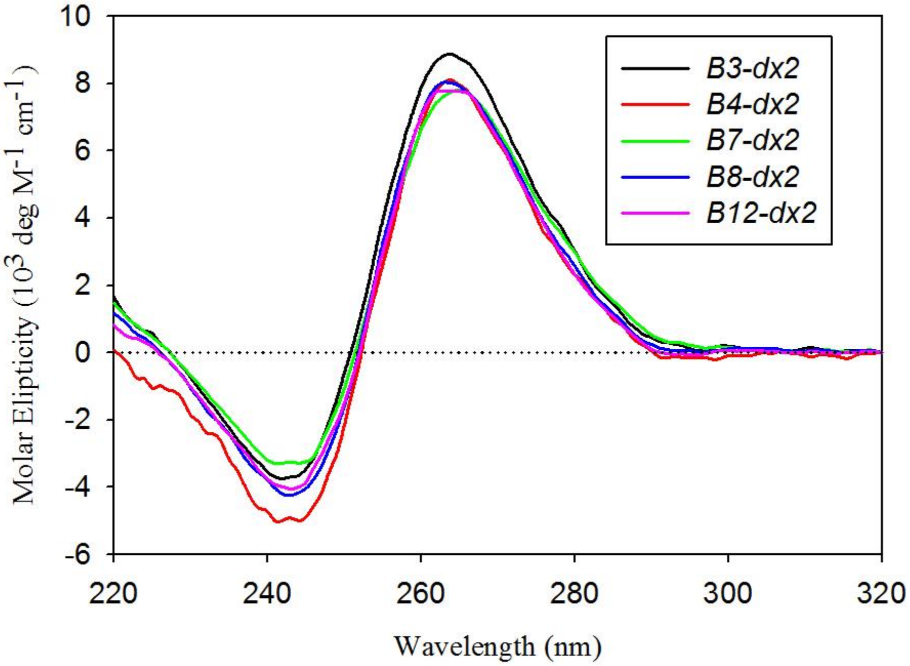

Figure S7. CD spectra of *B3-dx2* (black), *B4-dx2* (red), *B7-dx2* (green), *B8-dx2* (blue), and *B12-dx2* (pink) in buffer containing 30 mM KCl and 20 mM KPi (pH7).

References

1. Lim, K.W. and Phan, A.T. (2013) Structural basis of DNA quadruplex–duplex junction formation. *Angew Chem. Int. Ed. Engl.*, **52**, 8566–8569.
2. Russo Krauss, I., Spiridonova, V., Pica, A., Napolitano, V. and Sica, F. (2016) Different duplex/quadruplex junctions determine the properties of anti-thrombin aptamers with mixed folding. *Nucleic Acids Res.*, **44**, 983-991.
3. Huang, H., Suslov, N.B., Li, N.S., Shelke, S.A., Evans, M.E., Koldobskaya, Y., Rice, P.A. and Piccirilli, J.A. (2014) A G-quadruplex-containing RNA activates fluorescence in a GFP-like fluorophore. *Nat. Chem. Biol.*, **10**, 686-691.
4. Butovskaya, E., Heddi, B., Bakalar, B., Richter, S.N. and Phan, A.T. (2018) Major G-Quadruplex Form of HIV-1 LTR Reveals a (3 + 1) Folding Topology Containing a Stem-Loop. *J. Am. Chem. Soc.*, **140**, 13654-13662.
